# Supplementary material for: Stress-Inducible Gene Atf3 Dictates a Dichotomous Macrophage Activity in Chemotherapy-Enhanced Lung Colonization
Source: Int J Mol Sci. 2021 Jul 8;22(14):7356. doi: 10.3390/ijms22147356 (PMC8304704; doi:10.3390/ijms22147356)
Supplement: Supplementary file 1 [file ijms-22-07356-s001.zip › ijms-1216872-supplementary.pdf]

Supplementary Information for:
Stress-inducible gene *Atf3* dictates a dichotomous macrophage activity in chemotherapy-enhanced lung colonization

Justin D. Middleton[1], Jared Fehlman[1], Subhakeertana Sivakuma[1], Daniel G. Stover[2], and Tsonwin Hai[1*]

Department of Biological Chemistry and Pharmacology[1], Department of Internal Medicine[2], Ohio State University, Columbus, OH 43210, USA

Correspondence: Tsonwin Hai
Email: tsonwin.hai@osumc.edu

This PDF file includes:

Figures S1 to S9
Tables S1 to S2

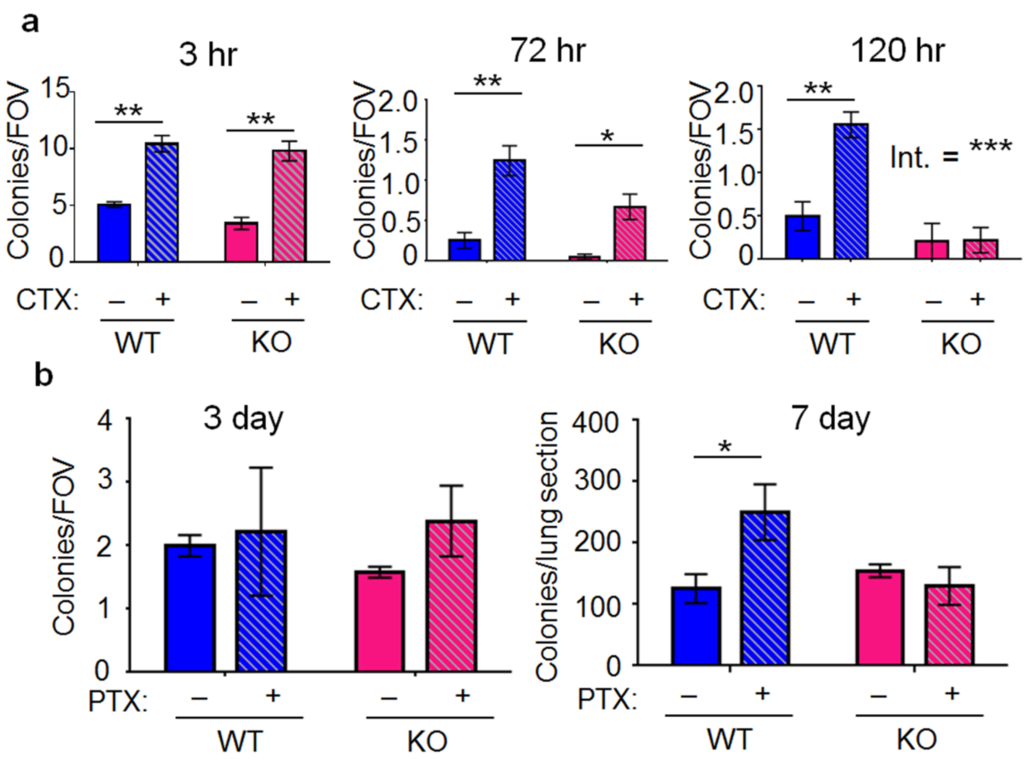

Figure S1. Chemotherapy exacerbated lung colonization in a host-*Atf3* dependent manner with multiple drugs and cancer cell lines. *(a)* FVB/N mice were treated with CTX (+) or vehicle (-), followed by an i.v. injection of the tGFP-Met1 breast cancer cells. Lungs were collected, stained by immunofluorescence for tGFP, and cancer colony per field of view (FOV) scored (n=3-4 for 3 hr, n=6-9 for 36 hr, n=3-4 for 120 hr, from 1-2 independent experiments). *(b)* C57BL/6 mice were treated with PTX (+) or vehicle (-), followed by i.v. injection of the PyMT breast cancer cells. Lungs were collected after 3 or 7 days, fixed, sectioned and stain with hematoxylin/eosin. Micromets were counted on a light microscope at 100X (n=3-4 mice/group). Bars indicate mean ± SEM; two-way ANOVA with post-hoc Holm-Šídák correction; Int.: treatment-genotype interaction; *$P<0.05$; **$P<0.01$.

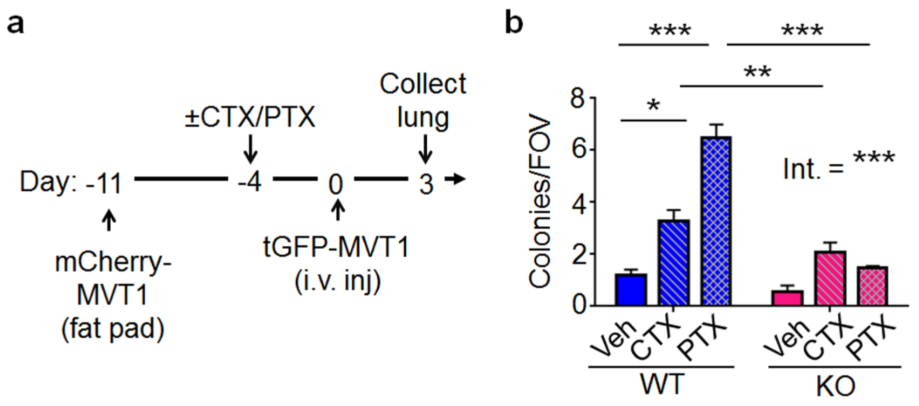

Figure S2. CTX and PTX exacerbated lung colonization in a host-*Atf3* dependent manner using a neoadjuvant coupled with double injection model. *(a)* A schematic of the model, where mice were injected with mCherry-MVT1 breast cancer cells at the mammary fat pad. Seven days later (when the primary tumor was palpable), mice were treated with PTX, CTX, or their respective vehicles (Veh), followed by i.v. injection of the tGFP-MVT1 breast cancer cells four days later. Lungs were collected 72 hr post-cancer cell injection. *(b)* Numbers of tGFP-MVT1 colonies per FOV (n=6-9 mice/group, from 2 independent experiments). Bars indicate mean ± SEM; two-way ANOVA with post-hoc Holm-Šídák correction; Int.: treatment-genotype interaction; **P*<0.05; ***P*<0.01; ****P*<0.001.

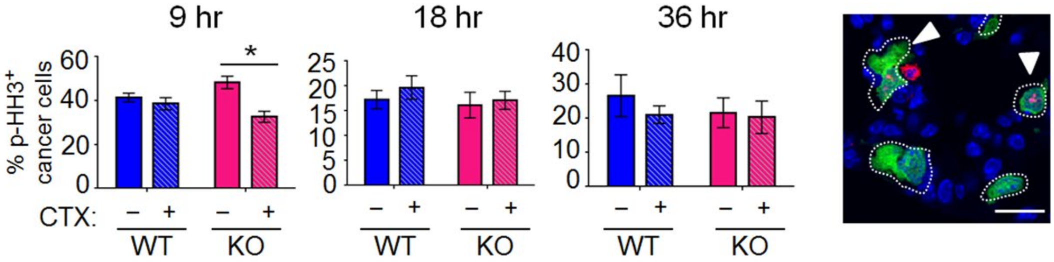

Figure S3. CTX and the host-*Atf3* had no major effect on cancer cell proliferation. Left: Percent (%) of tGFP$^+$ cancer cells positive for phospho-histone H3 (p-HH3, a proliferation marker) at the indicated time points (n=4-9 per time point, from 2 independent experiments). Minimum of 40 tGFP$^+$ cells were counted per lung section. Right: A representative image. Green: tGFP (cancer cells); red: phospho-histone H3; blue: Topro (nuclei). Dotted lines indicate cancer cells; arrowheads indicate proliferating cancer cells. Scale bar, 10 µm. Bars indicate mean ± SEM; two-way ANOVA with post-hoc Holm-Šídák correction; *P<0.05.

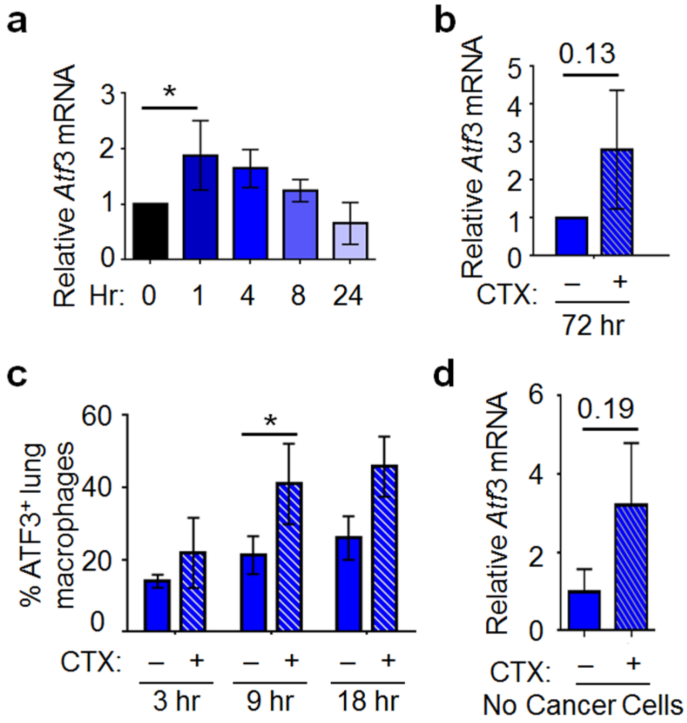

Figure S4. Induction of *Atf3* by CTX. *(a)* Wild type (WT) BMDMs were treated with 4-hydroperoxy cyclophosphamide(4-OOH) for the indicated time and analyzed for *Atf3* mRNAs by RT-qPCR. Signals were standardized against that of GAPDH and the average level in the non-treatment group (0 hr) was arbitrarily defined as 1 (n=4, from4 independent experiments). *(b)* CD11b^{+} Ly6G^{-} cells were isolated from the lungs at 72 hr after cancer cell injection of the WT mice with CTX (+) or vehicle (-) pre-treatment. RNA was isolated and the level of *Atf3* mRNAs analyzed by RT-qPCR. Signals were standardized against that of GAPDH and the average level in the control group (without CTX) was arbitrarily defined as 1 (n=4, from 4 independent experiments). *(c)* Immunofluorescence anlaysis of ATF3 and F4/80 (a macrophage marker) in the WT lung at the indicated time points. Y-axis indicates the % of ATF3^{+} macrophages in the lung (n=3-6 per time point, from 1 or 2 independent experiments, with minimum 50 macrophages counted per lung section). *(d)* CD11b^{+} cells were isolated from the blood of WT mice at 16 hr after CTX treatment and analyzed as in panel (b) (n=4-5, from 2 independent experiments). Bars indicate mean ± SEM; two-way ANOVA with post-hoc Holm-Šídák correction; *$P < 0.05$.

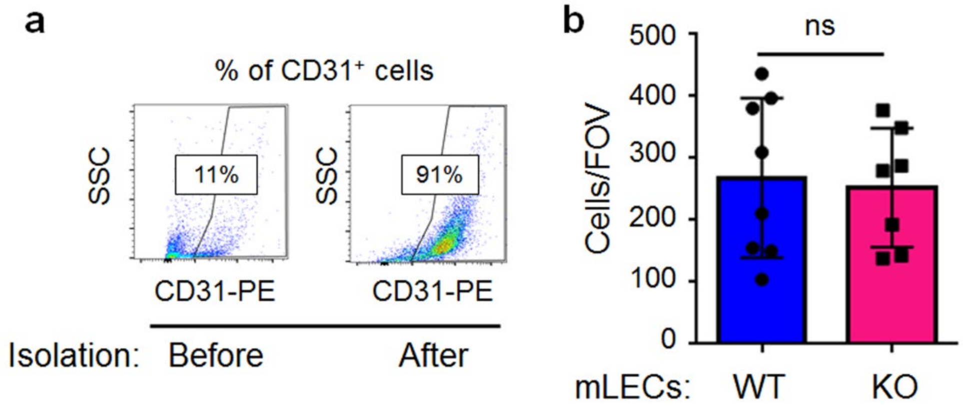

Figure S5. Analysis of mLECs purity and functionality. *(a)* Flow cytometry analysis of mLECs after magnetic bead isolation followed by two days in culture (to reach confluence). Single cell suspension from the lungs was analyzed as control (before isolation). *(b)* Data from transendothelial migration assays (Fig. 4b) are presented as cancer cells per FOV, showing that the genotype of mLECs did not affect cancer cell migration (n=7-8, from 2 independent experiments). Bars indicate mean ± SEM; two-way ANOVA with post-hoc Holm-Šídák correction; ns: not significant.

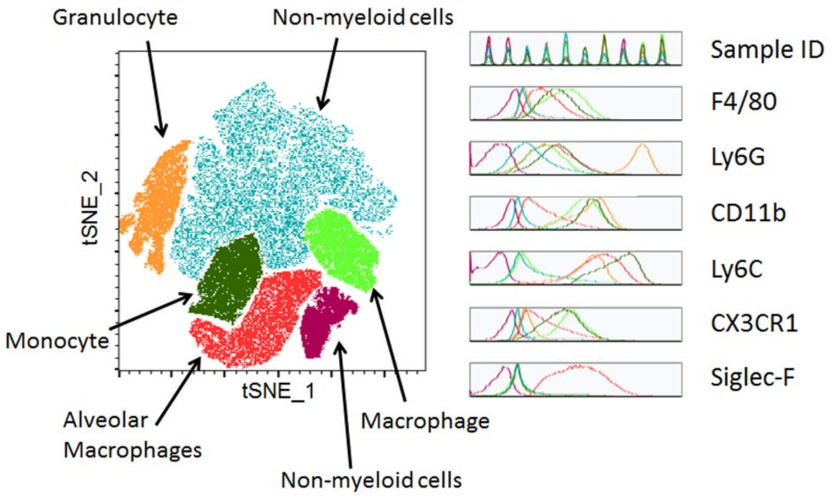

Figure S6. A representative image of tSNE analysis. Single cell suspensions of the lung at 72 hr after cancer cell injection were stained for CD11b, F4/80, Ly6G, Ly6C, CX3CR1 and Siglec-F. Flow data from four groups of lungs (*Atf3* KO with or without CTX pre-treatment, followed by clodronate or control liposome treatment as described in Fig. 5c legend, 3 mice per group) were concatenated into one overall sample and analyzed using a tSNE algorithm on Flow Jo software as described in Methods. Separate clusters were identified based on marker intensities on the right.

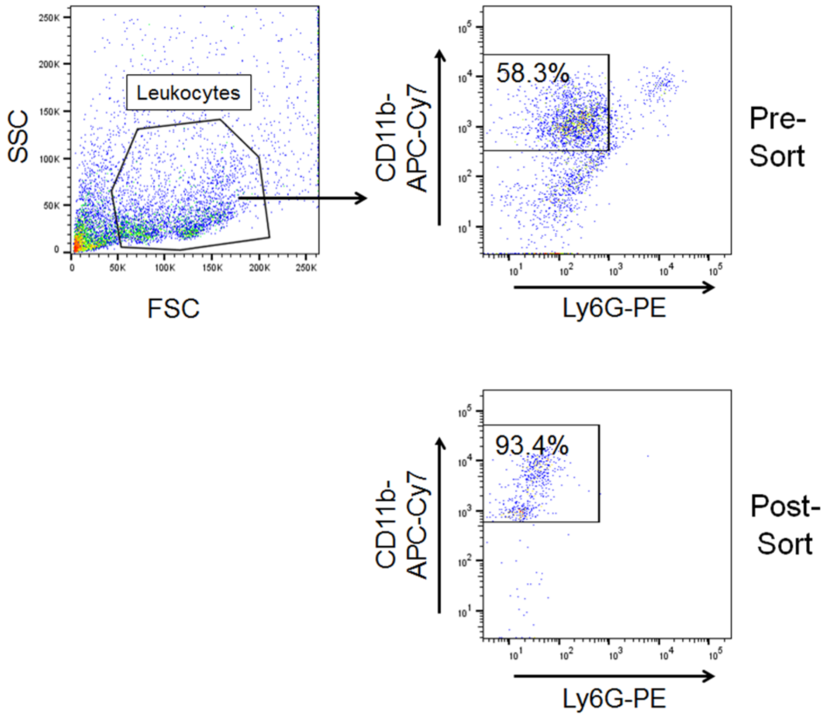

Figure S7. Representative images of lung monocyte/macrophage isolation via FACS. Flow analysis of the enriched monocyte/macrophage ($CD11b^+$, LyG^-) before and after sorting.

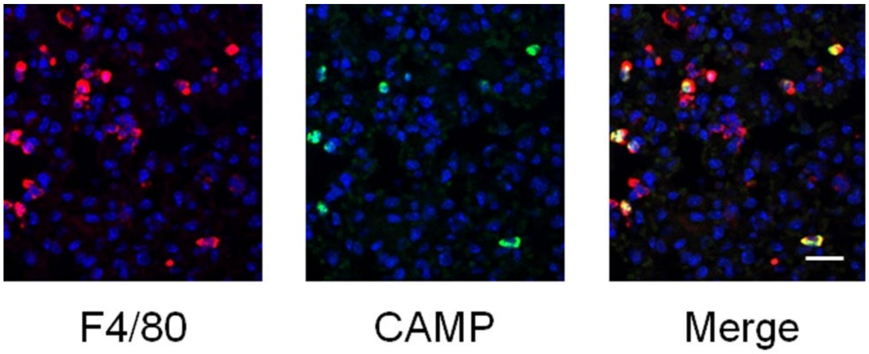

Figure S8. F4/80 and CAMP co-immunofluorescence analysis. Representative images of the *Atf3* KO lung with CTX pre-treatment, collected at 72 hr after cancer cell injection. Scale bar, 20 µm.

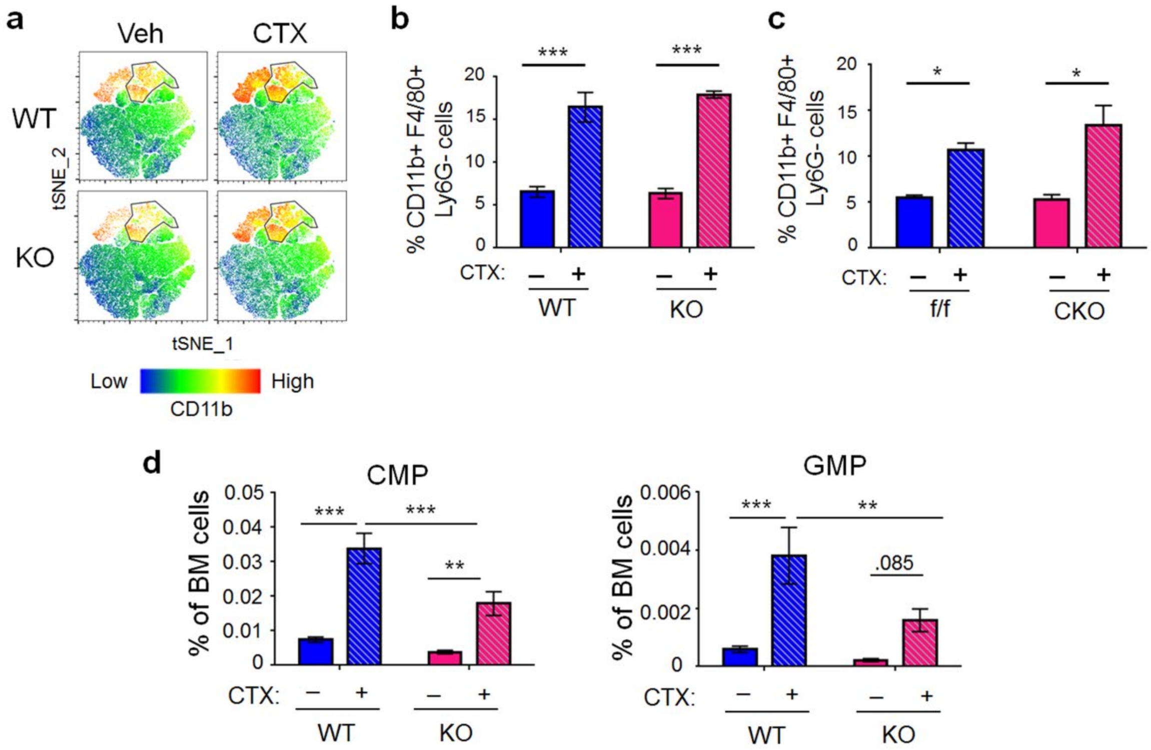

Figure S9. CTX increased macrophages in the lung. *(a)* A representative tSNE analysis of the lungs collected at 72 hr after cancer cell injection and stained for CD11b, F4/80, and Ly6G. The signal intensity for CD11b is shown and the black line encircles the $CD11b^+$, $F4/80^+$ LyG^- cells (macrophages). *(b)* Percent (%) of macrophages in the lungs from four groups of mice: WT or *Atf3* KO with CTX (+) or vehicle (-) pre-treatment (n=9-11, from 3 independent experiments). *(c)* Same as (b) except the *Atf3* CKO and control flox (f/f) mice were used (n=3, from 1 experiment). *(d)* % of the indicated myeloid progenitor cells in the bone marrow from four groups of mice: WT or Atf3 KO with CTX (+) or vehicle (-) pre-treatment at 5 days after CTX treatment (n=8-10, from 3 independent experiments). CMP: common myeloid progenitor cells (Lin^-, $Sca\text{-}1^-$, $Kit\text{-}1^+$, $IL7R^-$,$CD34^+$, $Fc\gamma R^-$), which give rise to all myeloid-lineage of cells; GMP: granulocyte-monocyte progenitors (Lin^-, $Sca\text{-}1^-$, $Kit\text{-}1^+$, $IL7R^-$, $CD34^+$, $Fc\gamma R^+$), which differentiate into monocytes, macrophages and granulocytes. Bars indicate mean ± SEM; two-way ANOVA with post-hoc Holm-Šídák correction; *$P<0.05$; **$P<0.01$; ***$P<0.001$.

Table S1. Primers used in this study

Target	Forward	Reverse
Atf3	5' -GAGATGTCAGTCACCAAGTC-3'	5'-CAGTTTCTCTGACTCTTTCTGC-3'
Camp	5’-CTTCAACCAGCAGTCCCTAGAC-3’	5’-GCCACATACAGTCTCCTTCACTC-3’
Lcn2	5’-TTTCACCCGCTTTGCCAAGT-3’	5’-GTCTCTGCGCATCCCAGTCA-3’
Ltf	5’-ACCGCAGGCTGGAACATC-3’	5’-CACCCTTCTCATCACCAATACAC-3’
Gapdh	5’-CAA CGG GAA GCC CAT CA-3’	5’-CGG CCT CAC CCC ATT T-3’

Table S2. Antibodies used in this study

**Name/Property**	**Assay**	**Dilution/Amount**	**Item #**	**Company**
anti-c-kit	Flow cytometry	0.125 μg/100 μl	48-1171-82	Invitrogen
anti-CD11b	Flow cytometry	0.125 μg/100 μl	47-0112-82	eBioscience
anti-CD16/CD32 Fc	Flow cytometry	0.5 μg /100 μl	14-0161-86	Invitrogen
anti-CD31	Flow cytometry	0.125 μg/100 μl	12-0311-82	eBioscience
anti-CD34	Flow cytometry	0.125 μg/100 μl	48-0341-82	Invitrogen
anti-CD64 (FcγR)	Flow cytometry	0.3 μg/100 μl	130-118-684	Miltenyi
anti-CX3CR1	Flow cytometry	0.125 μg/100 μl	149016	BioLegend
anti-F4/80	Flow cytometry	0.125 μg/100 μl	MCA497A488	Bio-Rad
Hematopoietic Lineage Antibody Cocktail	Flow cytometry	0.125 μg/100 μl	22-7770-72	Invitrogen
anti-IL7R	Flow cytometry	0.125 μg/100 μl	25-1271-82	Invitrogen
anti-Ly6C	Flow cytometry	0.125 μg/100 μl	48-5932-82	eBioscience
anti-Ly6G	Flow cytometry	0.125 μg/100 μl	17-9668-82	eBioscience
anti-NK1.1	Flow cytometry	0.125 μg/100 μl	12-5941-82	Invitrogen
anti-Sca-1	Flow cytometry	0.125 μg/100 μl	17-5981-83	Invitrogen
anti-Siglec-F	Flow cytometry	0.125 μg/100 μl	46-1702-82	Invitrogen
Anti-ATF3	IF	1:200	HPA001562	Atlas Antibodies
anti-CAMP	IF	1:200	NB100-98689	Novus Biologicals
anti-CD31	IF	1:50	ab28364	Abcam
anti-cleaved caspase-3	IF	1:100	9661S	Cell Signaling
anti-F4/80	IF	1:200	70076S	Cell Signaling
anti-phospho-histone-H3 (Ser 10)	IF	1:200	06-570	Sigma
anti-tGFP	IF	1:2000	PA5-22688	Invitrogen
CD31 MicroBeads	MACS	10 μl/100 μl	130-097-418	Miltenyi Biotec
CD11b MicroBeads	MACS	10 μl/100 μl	130-049-601	Miltenyi Biotec

IF: Immunofluorescence
